# Antiretroviral Drugs Regulate Epigenetic Modification of Cardiac Cells Through Modulation of H3K9 and H3K27 Acetylation

**DOI:** 10.3389/fcvm.2021.634774

**Published:** 2021-04-09

**Authors:** Shiridhar Kashyap, Avni Mukker, Deepti Gupta, Prasun K. Datta, Jay Rappaport, Jeffrey M. Jacobson, Steven N. Ebert, Manish K. Gupta

**Affiliations:** ^1^Division of Metabolic and Cardiovascular Sciences, Burnett School of Biomedical Sciences, College of Medicine, University of Central Florida, Orlando, FL, United States; ^2^Division of Pathology, Tulane National Primate Research Center, Covington, LA, United States; ^3^Department of Medicine, Center for AIDS Research, Case Medical Center, Case Western Reserve University and University Hospital, Cleveland, OH, United States

**Keywords:** antiretroviral therapy, cardiovascular disease, histone deacetylase, human immunodeficiency virus, methyltransferase, SIRT1, cellular hypertrophy, ROS

## Abstract

Antiretroviral therapy (ART) has significantly reduced the rate of mortality in HIV infected population, but people living with HIV (PLWH) show higher rates of cardiovascular disease (CVD). However, the effect of antiretroviral (ARV) drug treatment on cardiac cells is not clear. In this study, we explored the effect of ARV drugs in cardiomyocyte epigenetic remodeling. Primary cardiomyocytes were treated with a combination of four ARV drugs (ritonavir, abacavir, atazanavir, and lamivudine), and epigenetic changes were examined. Our data suggest that ARV drugs treatment significantly reduces acetylation at H3K9 and H3K27 and promotes methylation at H3K9 and H3K27, which are histone marks for gene expression activation and gene repression, respectively. Besides, ARV drugs treatment causes pathological changes in the cell through increased production of reactive oxygen species (ROS) and cellular hypertrophy. Further, the expression of chromatin remodeling enzymes was monitored in cardiomyocytes treated with ARV drugs using PCR array. The PCR array data indicated that the expression of epigenetic enzymes was differentially regulated in the ARV drugs treated cardiomyocytes. Consistent with the PCR array result, SIRT1, SUV39H1, and EZH2 protein expression was significantly upregulated in ARV drugs treated cardiomyocytes. Furthermore, gene expression analysis of the heart tissue from HIV+ patients showed that the expression of SIRT1, SUV39H1, and EZH2 was up-regulated in patients with a history of ART. Additionally, we found that expression of SIRT1 can protect cardiomyocytes in presence of ARV drugs through reduction of cellular ROS and cellular hypertrophy. Our results reveal that ARV drugs modulate the epigenetic histone markers involved in gene expression, and play a critical role in histone deacetylation at H3K9 and H3K27 during cellular stress. This study may lead to development of novel therapeutic strategies for the treatment of CVD in PLWH.

## Introduction

Human immunodeficiency virus (HIV) has infected 38 million people globally, and 1.7 million new cases were diagnosed in 2019 (1). Although ART improves the life expectancy of people living with HIV (PLWH), it is also known to increase the risk for developing cardiovascular disease (CVD) (2–5). Recent studies have shown that cardiovascular risk is almost double in HIV patients compared to the healthy population, and claims more lives of HIV patients compared to any other disease (6–8). Many HIV patients began taking ARV drugs in the mid 1990's and are now reaching the age of 50, which suggests that the association of HIV infection and cardiovascular disease was established three decades ago (9, 10). It is estimated that by year 2030, 73% of HIV infected patients will show CVD by the age of 50 (11).

Due to application of ART, viral replication is inhibited and viral load becomes undetectable in the patient's serum. However, the limitation of ARV drugs is that patients have to commit to this treatment for their entire life to control viral reactivation. People receiving ART live longer, but often show symptoms of organ failure, neuronal dementia, kidney failure, and aging, (12–14). The first ART was the nucleoside reverse transcription inhibitor (NRTI) azidothymidine (AZT), which produced severe organ toxicity (15). Later, several less-toxic reverse transcriptase inhibitors, such as lamivudine, were developed (16). Due to partial effectiveness of the NRTI drugs, in 1995 FDA approved the use of another class of antiretroviral drug protease inhibitor in combination of NRTIs (17). In general, patients receive a combination of two or three drugs, such as nucleoside reverse transcriptase inhibitors (lamivudine and abacavir), along with protease inhibitors (ritonavir and atazanavir) (18). Although, in 2020 international antiviral society- USA panel suggest that regimens of three drugs including 2 nucleoside reverse transcriptase inhibitor and an integrase inhibitor can be useful to suppress the viral replication (19), some of the developing nations continuously using the old regime of first line NRTI- based cocktail along with protease inhibitors (20–22). Chronic administration of multiple drugs may lead to CVD and heart failure in HIV patients through cellular and molecular modification in cardiomyocytes, which leads to modulation of pathological gene expression (5, 23, 24). Concordant with the clinical data, it was also reported that combined ART causes cardiomyopathy and metabolic disorders in HIV mouse models (25).

Post translational modifications (PTMs) of histone protein are responsible for epigenetic changes, which regulate DNA conformation and chromatin packaging, leading to activation and/or suppression of associated gene expression (26, 27). Epigenetic changes in histones (acetylation and methylation) play a significant role in maintaining cellular homeostasis during environmental and oxidative stress (28, 29). ART is known to inhibit function of the endoplasmic reticulum and mitochondria, and induces oxidative stress (30), which may initiate modulation of epigenetic signatures and gene expression. These global epigenetic changes may impact transcriptional regulation of protective or detrimental gene expression in cardiomyocytes, leading to heart failure (31, 32). Acetylation of histone 3 at lysine 9 and 27 (H3K9ac and H3K27ac) promotes active gene transcription (33–35), whereas acetylation at H3K9 in the promoter of cardiac specific transcription factors induces expression of fatal genes that lead to cardiac hypertrophy (36). The epigenetic regulatory enzyme Sirtuin 1 (SIRT1) acts as a deacetylase at H3K9 marks of histone and regulates cellular oxidative stress through inactivation of fatal gene expression (37–39). Additionally, SIRT1 protects cells during stress through suppression of reactive oxygen species (ROS) production and reduction of cellular hypertrophy (37, 40).

This study was designed to explore the epigenetic relationship of ARV drugs with histone markers and their regulatory enzymes. We found that ARV drugs treatment led to decreased acetylation of histone 3 (at H3K9 and H3K27) and increased tri-methylation (at H3K9 and H3K27). Additionally, we found that ARV treatment modulates the expression of epigenetic enzymes specific to histone acetylation and methylation that may help to maintain cellular homeostasis during drug induced stress.

## Methodology

### Human Subject's Ethics Statement

Written informed consent was obtained from the participant individuals by National NeuroAIDS Tissue Consortium (NNTC) (New York), and partnering institute according to local IRB protocol. Human heart tissue from HIV positive patients and healthy donors were obtained from NNTC according to approved IRB protocol by University of Central Florida.

### Cell Models and ARV Drugs Treatment

Animal studies were approved by the institutional IACUC. All experiments were performed with neonatal rat ventricular cardiomyocytes (NRVCs). Primary cardiomyocytes were isolated from the 1–2-day-old Harlan Sprague–Dawley rats (Jackson Laboratory, Bar Harbor, ME) as described previously (41). In brief, left ventricles were collected and digested with 0.05% trypsin at 4°C overnight, followed by collagen treatment for 40 min at 37°C. Cardiomyocytes were separated by pre-plating the digested cell suspension. Initially, NRVCs were grown in MEM (Gibco, Grand Island, NY) containing 10% fetal bovine serum (FBS) with 1X anti-anti (Gibco) for 24 h in 10 cm plates for protein isolation, and 2-well-chamber slides for immunostaining at a density of 1.5 × 10^6^ and 1 × 10^5^ cells, respectively. Total RNA was isolated from 1 × 10^5^ cells grown in 6-well-plates. All experiments were performed in NRVCs grown for least 12 h in DMEM with 2% FBS. For western blot and microscopy, cells were treated with a combination of 5 μM each of the ARV drugs ritonavir, abacavir, atazanavir, and lamivudine for 4, 12, or 24 h (Selleck Chemicals Llc, Pittsburgh, PA). For adenovirus mediated overexpression, cells were incubated with adenovirus (1:1 pfu) in serum free DMEM for 2 h, then the cells were incubated in DMEM with 2% FBS for 48 h. The adenoviruses encoding green florescence protein (Ad-GFP), EZH2 (Ad-EZH2), and SIRT1(Ad-SIRT1) were obtained from Vector Biolabs (Malvern, PA).

### PCR Array Profiling for Chromatin Modifying Enzymes

Total RNA was isolated from NRVCs treated with ARV drugs using the RNeasy miniprep kit (Qiagen, Germantown, MD) following the manufacturer instructions and quantified by Nanodrop 8000 (Thermo Scientific, Waltham, MA). Epigenetic modifying enzyme expression was assessed using the rat chromatin RT^2^ Profiler PCR array (PARN-085Z, Qiagen). First-strand cDNA was generated from 500 ng total RNA using the RT^2^ first-strand synthesis kit (Qiagen). Quantitative real-time PCR (qRT-PCR) reactions were prepared with RT^2^ SYBR Green/ROX PCR master mix and run on the Step One-Plus PCR machine (Applied Biosciences, Foster City, CA) with the standard SYBR green PCR program. PCR data was analyzed using the web-based tool provided by manufacturer (https://dataanalysis2.qiagen.com/pcr). Differentially expressed chromatin modifying enzymes in ARV drugs-treated NRVCs were identified with a fold change >1.2. Statistical significance was determined by Student's *t*-test of the replicate 2^−ΔΔCT^ values for each gene in the control vs. treatment groups. A *p* < 0.05 was considered significant. PCR array results were normalized using the housekeeping genes Actb, Ldha, Bm2, and Hrpt1. Expression of differentially expressed epigenetic chromatin modification enzymes was validated by qRT-PCR and western blot.

### Expression Analysis by Real-Time PCR

RNA from cardiomyocytes was isolated using the RNeasy Mini kit (Qiagen). TRI reagent (Sigma, St. Louis, MO) was used for RNA isolation from clinical tissue samples and treated with RNase free DNAse (Qiagen) to remove DNA contamination. cDNA was synthesized with 500 ng RNA using a SuperScript III First-Strand Synthesis SuperMix reagent kit (ThermoFisher Scientific). Gene expression was analyzed by qRT-PCR using SYBR Green master mix (Applied Biosystems) using gene specific primers (Table 1). Data were normalized with GAPDH as internal control.

**Table 1:** Primer set used for genes expression analysis.

**Gene**	**Sequence (5**^**′**^**-3**^**′**^**) Forward**	**Sequence (5**^**′**^**-3**^**′**^**) Reverse**
SIRT1 (Rat)	CTCCCAGATCCTCAAGCCAT	GCTCATGAATGCTGAGTTGCT
SUV39H1 (Rat)	GGGGTTGCTCTAGAATGTGGT	ATAAGGGGCCCCAAGTAGGA
EZH2 (Rat)	TCTCACCAGCTGCAAAGTGT	ACAAGTGACTCAACAACAAGTTCA
Suv39h1 (Human)	TGATGAGGGGCGGATTGAAC	CCGTAACCACGTACAGCCAT
Ezh2 (Human)	GGACTCAGAAGGCAGTGGAG	CTTCCGCCAACAAACTGGTC
SIRT1 (Human)	CCCTCAAAGTAAGACCAGTAGC	CACAGTCTCCAAGAAGCTCTAC
GAPDH (Human)	GTCTCCTCTGACTTCAACAGC	ACCACCCTGTTGCTGTAGCCAA

### Protein Extraction and Western Blot Analysis

For western blotting, NRVCs were lysed with RIPA buffer (50 mM tris-HCl pH-8.0, 150 mM NaCl, 1% IGEPAL, 12 mM sodium deoxycholate, 1% SDS) and 1X mammalian protease inhibitor (Sigma). Cells were sonicated for 24 s with a setting for 2 s. on and 1 s. off at 35% amplitude (Q125 sonicator, QSonica, New Orleans, LA). Cell debris and unbroken cells were removed by centrifuged at 10,000 × g for 10 min at 4°C and supernatants were collected for western blotting. Protein concentration was measured using the BCA assay kit (ThermoFisher Scientific). Protein samples were prepared in 1X Laemmli buffer and resolved by SDS-PAGE (Bio-Rad, Hercules, CA). Resolved proteins were then transferred to PVDF membrane by electrophoresis. Membranes were blocked with LI-COR blocking buffer (LI-COR, Lincoln, NE) for 1 h at room temperature and incubated overnight at 4°C with primary antibody in blocking buffer (LI-COR). The membranes were then probed with secondary antibody (IRDye^®^680, red and 800, green, LI-COR) at room temperature for 2 h after washing twice with 1X PBST and once with 1X PBS. After incubation, the membranes were washed twice with 1X PBST and once with 1X PBS before scanning using a LI-COR-Odyssey scanner (LI-Cor). The following antibodies were used for western blotting: SIRT1, Histone, Suv39h1, H3K9me1, H3K9me2, H3K27me1, H3K9me2 (Cell Signaling, Danvers, MA), H3K9ac (MyBioSource, San Diego, CA), H3k27ac, H3K9me3, EZH2 (Abcam, Cambridge, MA), and GAPDH (Proteintech, Rosemont, IL).

### Cell Viability Assay

Cellular viability was detected using CellTiter-Glo (Promega, Madison, WI). NRVCs were plated in a 96-well white plate at a density of 10,000 cells per well. The cells were then treated with ARV drugs (5 μM of Ritonavir, Abacavir, Atazanavir and Lamivudine, (Selleck Chemicals Llc, Pittsburgh, PA) for 24 h. Cellular viability was detected using the CellTiter-Glo luminescent cell viability assay (Promega). The plate was scanned with an EnVision luminometer (PerkinElmer, Waltham, MA) for a measurement time of 2 s.

### Immunostaining

For immunocytochemistry, NRVCs plated in chamber slides were washed twice with 1X PBS and then fixed with 4% paraformaldehyde (PFA) for 10 min. After washing twice with 1X PBS, cells were permeabilized with 0.5% Triton X-100 for 10 min at room temperature. Cells were masked with 0.1 M glycine for 30 min at room temperature and then washed twice with 1X PBS. Blocking was performed at room temperature with blocking buffer (1% BSA, 0.1% Tween 20 in 1X PBS) for 1 h, then the slides were incubated with primary antibody in blocking buffer overnight at 4°C. After washing with 1X PBS, cells were probed with secondary antibody labeled with Alexa Fluor 488 and 490 (Thermo Fisher Scientific) in blocking buffer for 1 h at room temperature. For counter staining with a second primary antibody, cells were blocked for additional 30 min at room temperature before probing with antibody. Cells were mounted with VECTASHIELD HardSet mounting medium with DAPI (Vector Laboratories, Burlingame, CA). Images were captured with a Zeiss 710 fluorescence microscope (Oberkochen, Germany). The following antibodies were used for immunocytochemistry H3K9ac (MyBioSource,), Actinin, H3K27ac, H3K9me3, H3K27me3, Ezh2 (Abcam), SIRT1 (Cell signaling).

### ROS Measurement

Intracellular ROS level were detected in live cells by dihydroethidium (DHE) florescence probe (Life Technology) staining. NRVCs were plated in the 35 mm plate with DMEM having 2% FBS for the DHE staining. Cardiomyocytes were treated with cocktail of 5 μM each of the ARV drugs ritonavir, abacavir, atazanavir, and lamivudine or DMSO for 24 h and then live cells were incubated with DHE probe (5 uM) with cell culture media for 5 min. Then cells were washed with the culture media and further incubated in the cell culture media for the microscopy. DHE fluorescence intensity was measured using fluorescence microscope (BZ-X800, Keyence, Osaka, Japan).

### Knockdown of SIRT1 and Ezh2

For knockdown of SIRT1, 1 × 10^6^ rat primary cardiomyocytes were plated in 60 mm plates. siRNAs were transfected using Lipofectamine 2000 in optimum media (Gibco) for 3 h, then the media was replaced with DMEM having 2% FBS for 48 h. Sirt1 was knocked down in rat cardiomyocytes using siRNA (SASI-Rn 02; 00230695, 00230696, 00230697, Sigma), and control cells were transfected with universal negative siRNA (Sigma). When needed, cells were incubated with ARV drugs (5 μm of ritonavir, abacavir, atazanavir, and lamivudine) in DMEM having 2% FBS for different time points and cells were harvested for protein as described above. Similarly, knockdown of Ezh2 was performed using siRNA (s155284, Thermofisher).

### SIRT1 Deacetylase Activity Assay

SIRT1 mediated deacetylase activity was measured using the SIRT1 activity assay kit (Abcam) according to the manufacturer instructions. Cell lysate was prepared in lysis buffer as described in the protocol. The reaction mixture was prepared by mixing SIRT1 assay buffer, fluoro-substrate peptide, and developer. The reaction was initiated by adding 15 μg protein lysate and incubated for 30 min in black transparent bottom 96-well-plates (Thermo Scientific). The fluorescent intensity was measured by spectrophotometer (Bio-Tak, Synergy 4, Winooski, VT) at 350 nm excitation and 450 nm emission, respectively.

### Data Analysis and Statistical Procedures

All the experiments were performed three or more times. Statistical analyses were performed using Prism GraphPad 8.0. The results are presented as mean ± standard deviation. The unpaired student *t*-test was performed for statistical significance between the control and test groups. A *p* < 0.05 was considered to be significant.

## Results

### ARV Drugs Suppress Histone Marks Responsible for Activation of Gene Expression

**Figure 1:**
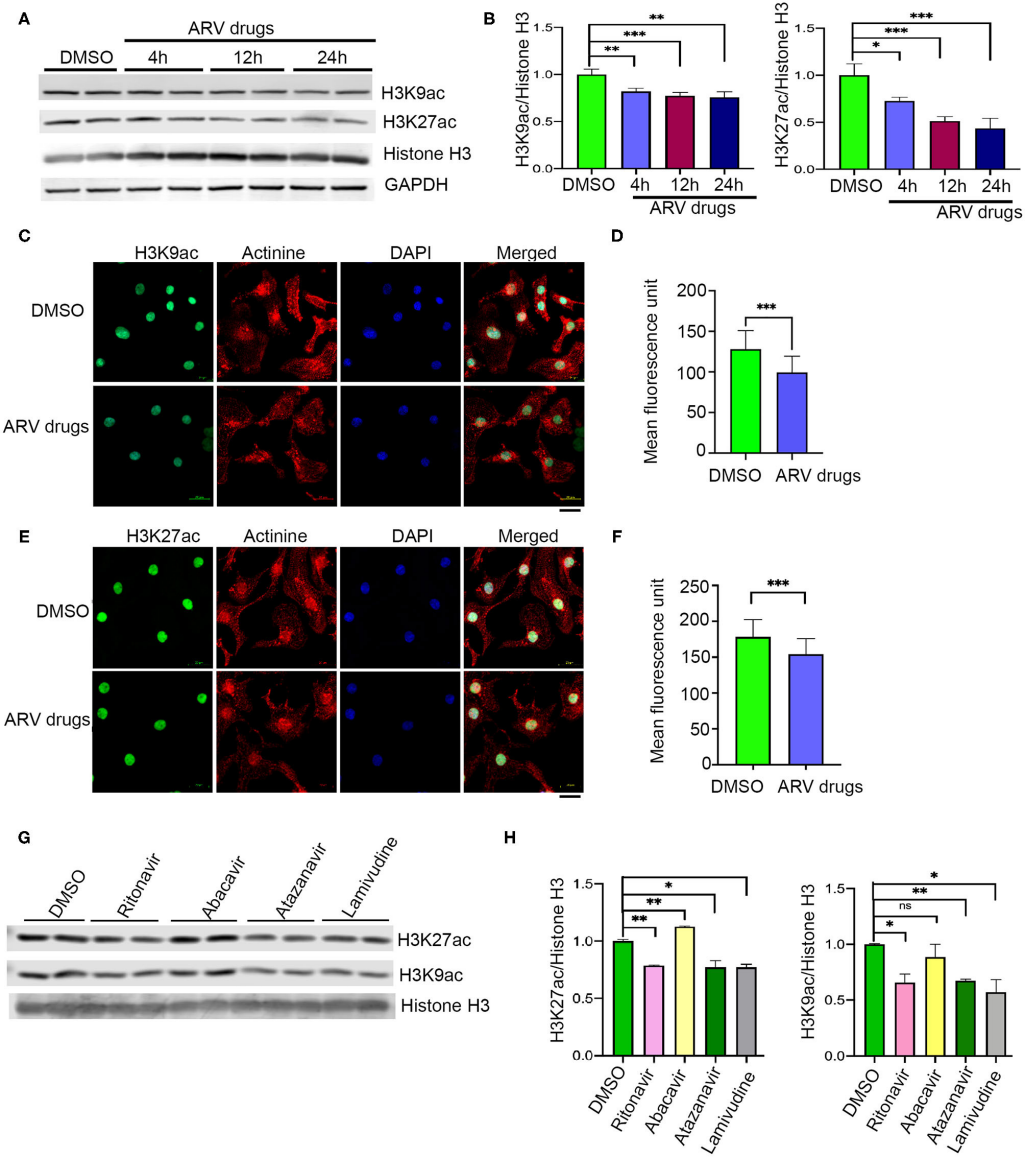
ARV drugs treatment suppresses active histone marks. **(A,B)** Western blots show acetylation of histone at H3K9 and H3K27 and graphs show its quantification (^***^p < 0.001). NRVCs were treated with ARV drugs (5 μM of Ritonavir, Abacavir, Atazanavir and Lamivudine) for 4, 12, and 24 h and western blots were done with total protein lysate. **(C,D)** Representative images show immunofluorescence staining of NRVCs stained with H3K9ac (green), actinin (red), and nucleus stained with DAPI (blue). Cells were treated with ARV drugs for 12 h and fixed with 4% PFA. **(D)** Graph shows quantification of microscopic images (*n* = 50 cells); (^*^p < 0.05). **(E,F)** Representative images show immunofluorescence staining of NRVCs stained with H3K27ac (green), actinin (red), and nucleus stained with DAPI (blue). Cells were treated with ARV drugs for 12 h and fixed with 4% PFA. **(D)** Graph shows quantification of microscopic images (*n* = 50 cells); (^*^p < 0.05). **(G,H)** Western blot shows acetylation of NRVCs after drugs treatment. NRVCs were treated with individual ARV drug for 12 h and western blot was done with total protein lysate. Graphs show quantification of western blot (^***^p < 0.001; ^**^p < 0.01, ns, not significant).

**Figure 2:**
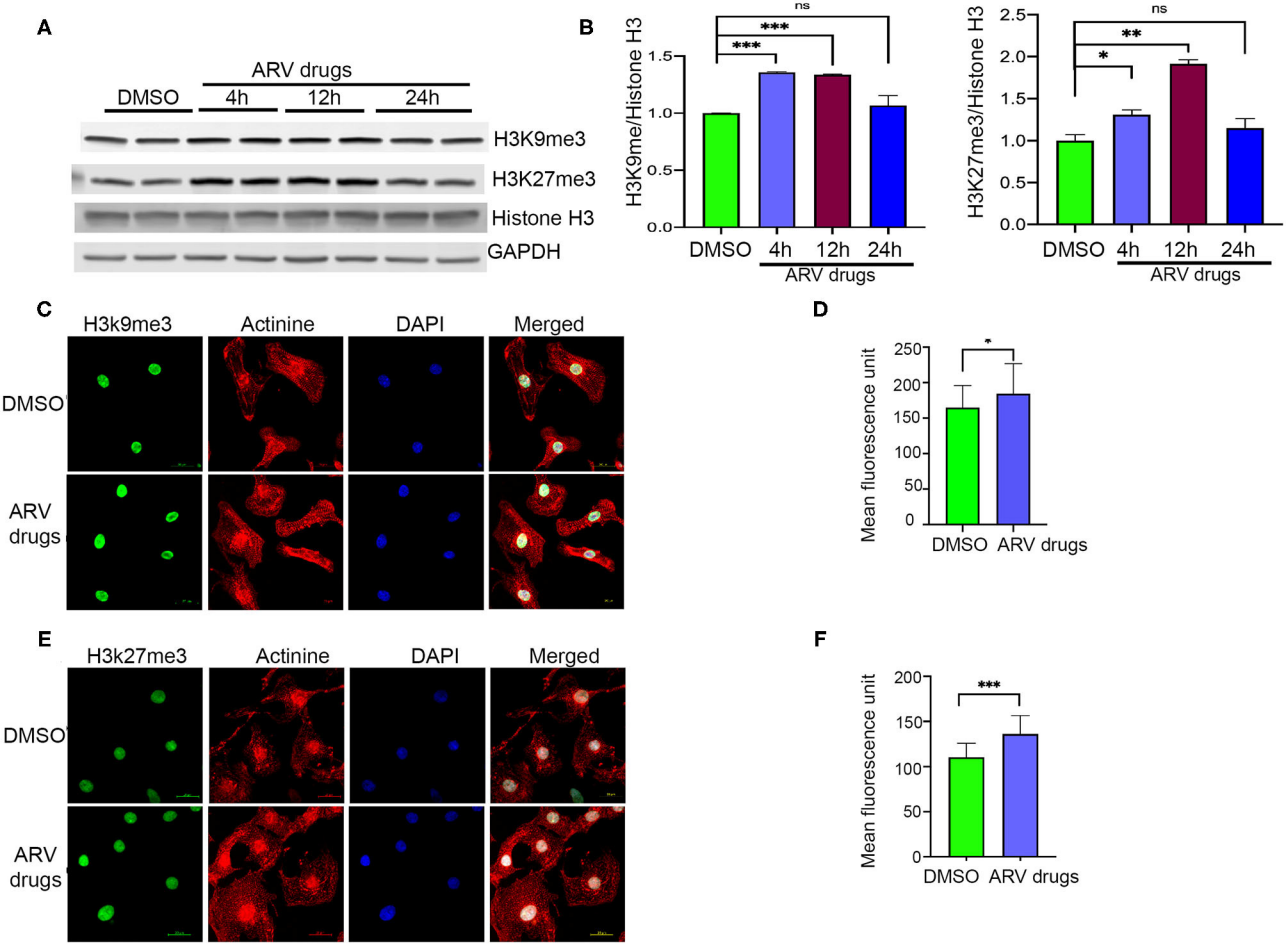
ARV drugs treatment promotes repressive histone marks. **(A,B)** Western blot shows antiretroviral drug treatment increase the methylation of H3K27me3 and H3K9me3. NRVCs were treated with ARV drugs (5 μM of Ritonavir, Abacavir, Atazanavir and Lamivudine) for 4, 12, and 24 h and western blots were done with total protein lysate. Graph shows quantification of western blots (^***^p < 0.001; ^**^p < 0.01, ns, not significant) **(C)** Representative images show immunofluorescence staining of NRVCs stained with H3K9me3 (green), actinin (red), and nucleus stained with DAPI (blue). Cells were treated with ARV drugs for 12 h and fixed with 4% PFA. **(D)** Graph shows quantification of microscopic images (*n* = 50 cells); (^*^p < 0.05). **(E)** Representative images show immunofluorescence staining of NRVCs stained with H3K27me3 (green), actinin (red), and nucleus stained with DAPI (blue). Cells were treated with ARV drugs for 12 h and fixed with 4% PFA. **(F)** Graph shows quantification of microscopic images (*n* = 50 cells); (^***^p < 0.001).

Earlier studies suggested that PTMs of histones can influence biological processes through activation or deactivation of cellular genes. Histone deacetylase regulates acetylation levels at H3K9 and H3K27, and plays a critical role in promoting chromatin inactivation marks (42–44). Another epigenetic regulating enzyme, histone methyltransferase regulates cellular gene deactivation by promoting tri-methylation of histones at H3K9 and H3K27 (45, 46). The effect of ARV drugs on the regulation of epigenetic histone marks is unclear. In this study, we tested the role of ARV drugs on the expression of epigenetic regulatory enzymes and its effect on cardiac epigenetic remodeling. To understand the effects of drugs on histone PTMs, NRVCs were treated with plasma levels of ARV drugs (5 μM of ritonavir, atazanavir, abacavir, and lamivudine) (47–50) for different duration (4, 12, and 24 h), and the level of histone acetylation and methylation was determined by western blotting. Western blot analysis showed that ARV drugs treatment significantly reduced acetylation at H3K9 and H3K27 (Figures 1A,B). To confirm ARV drugs mediated changes in histone PTMs, immunocytochemistry was performed using NRVCs and images were captured by confocal microscopy. Microscopy data showed that drug treatment significantly reduced acetylation of histone 3 at K9 (Figures 1C,D) and K27 (Figures 1E,F). We further tested the effects of individual drugs on histone protein acetylation. NRVCs were treated with individual ARV drugs for 12 h and the level of histone acetylation was detected by western blot. Western blot analysis showed that the protease inhibitors (ritonavir, atazanavir) and reverse transcriptase inhibitor lamivudine had a greater deacetylation effect compared to the reverse transcriptase inhibitor abacavir (Figures 1G,H) These results suggest that ARV drugs modulate PTMs of histone and cause significant reduction of active histone marks. Earlier studies reported that mono, di, and tri-methylation of histone plays a significant role in the regulation of chromatin mediated gene expression in heart (29, 31, 32). In this study, we tested the expression of histone methylation in cardiomyocytes after drug treatment. Western blot data indicated that drug treatment significantly increased tri-methylation at H3K9 and H3K27 histone marks (Figures 2A,B). Further immunocytochemistry was done in ARV treated cardiomyocytes with the H3K9me3 and H3K27me3 antibodies to evaluate status of histone 3 methylation. Microscopy data suggested that ARV drugs treatment significantly upregulate methylation of histone protein at H3K9 (Figures 2C,D) and H3K27 (Figures 2E,F). Additionally, we performed western blot analysis to determine the level of mono and dimethylation of histone in ARV drugs treated cells. Western blot analysis showed that ARV drugs treatment significantly increased the level of dimethylated histone (H3K9me2, H3K27me2) in drug treated cells but not the monomethylated form of histone (H3K9me1, H3K27me1) (Supplementary Figures 1A,B). To determine the effect of ARV drugs on cardiomyocytes remodeling and pathology, we treated the NRVCs with combination drugs for 24 h and cellular hypertrophy and ROS level were determined. Our microscopy data show that ARV drugs treatment significantly increases the cardiomyocytes cell size (Supplementary Figures 2A–C), which is an indicator of cellular hypertrophy. Additionally, we found that ARV drugs treatment significantly increases the cellular ROS level (Supplementary Figures 2D–F). The increased ROS production is known to cause cardiomyocytes remodeling and pathological changes during stress condition.

### Chromatin Modifying Enzymes Play a Critical Role in Epigenetic Modification of Histone During ARV Drugs Treatment

**Figure 3:**
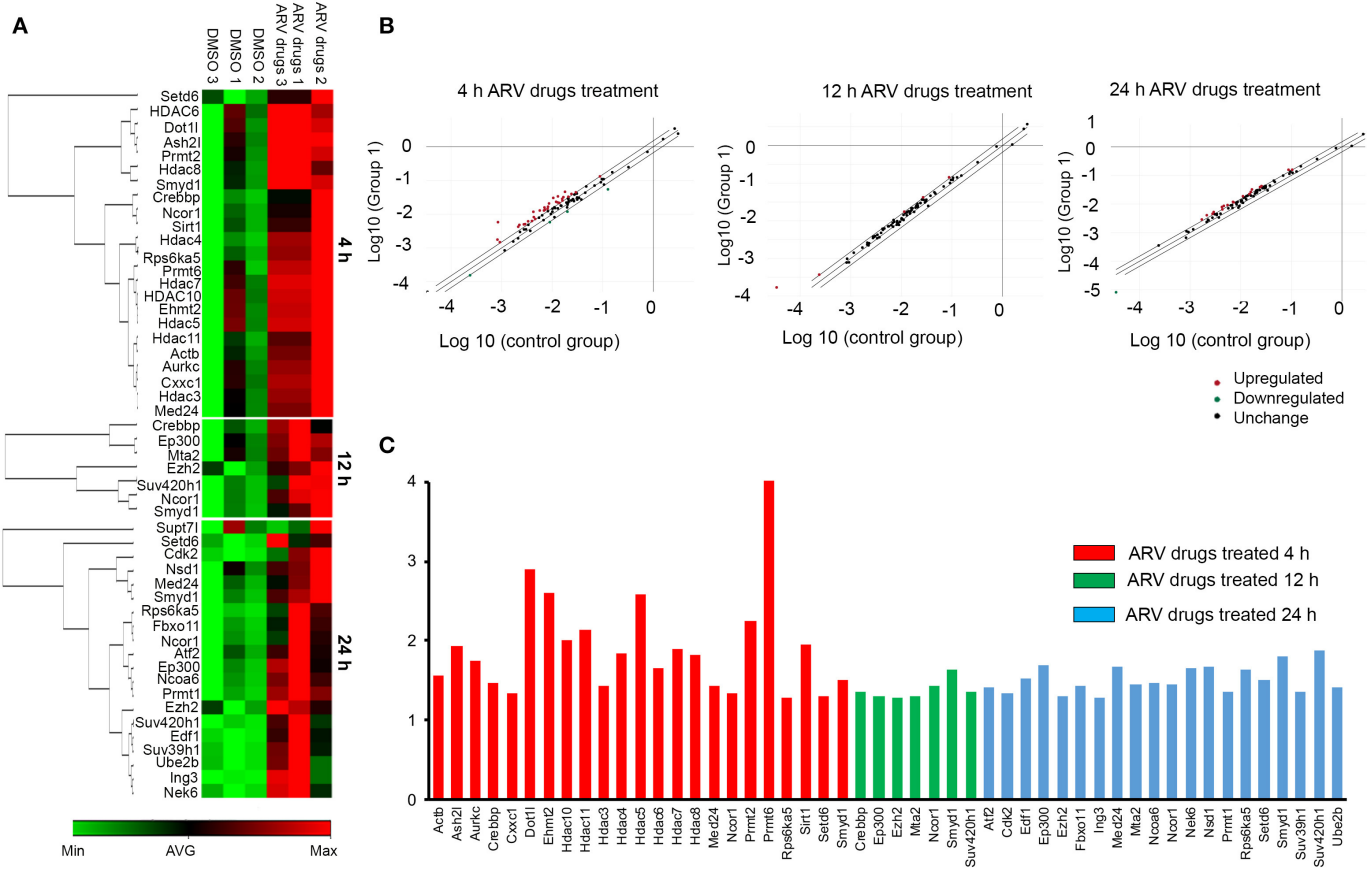
ARV drugs treatment modulates expression of epigenetic regulating enzyme. NRVCs were treated with ARV drugs (5 μM of Ritonavir, Abacavir, Atazanavir, and Lamivudine) for 4, 12, and 24 h and expression of epigenetic enzyme was measured by RT^2^ PCR array profiling. **(A)** Clustergram showing differentially expressed genes (*p* < 0.05, Fold Change >1.2) obtained on RT^2^ PCR array profiling, green represent minimum and red represent maximum magnitude of expression. **(B)** Scatter Plot representing normalized expression of genes between 4, 12, and 24 h ARV drugs treated vs. control group. Central diagonal line represents no change, whereas, outer diagonal lines indicate the fold regulation threshold (>1.2). Genes with data points beyond the outer lines in the upper left and lower right corners are up-regulated or down-regulated, respectively. **(C)** Graphical representation of differentially expressed chromatin modifying enzymes against expression regulation on drug treatment at 4 h (red), 12 h (green), and 24 h (blue).

**Figure 4:**
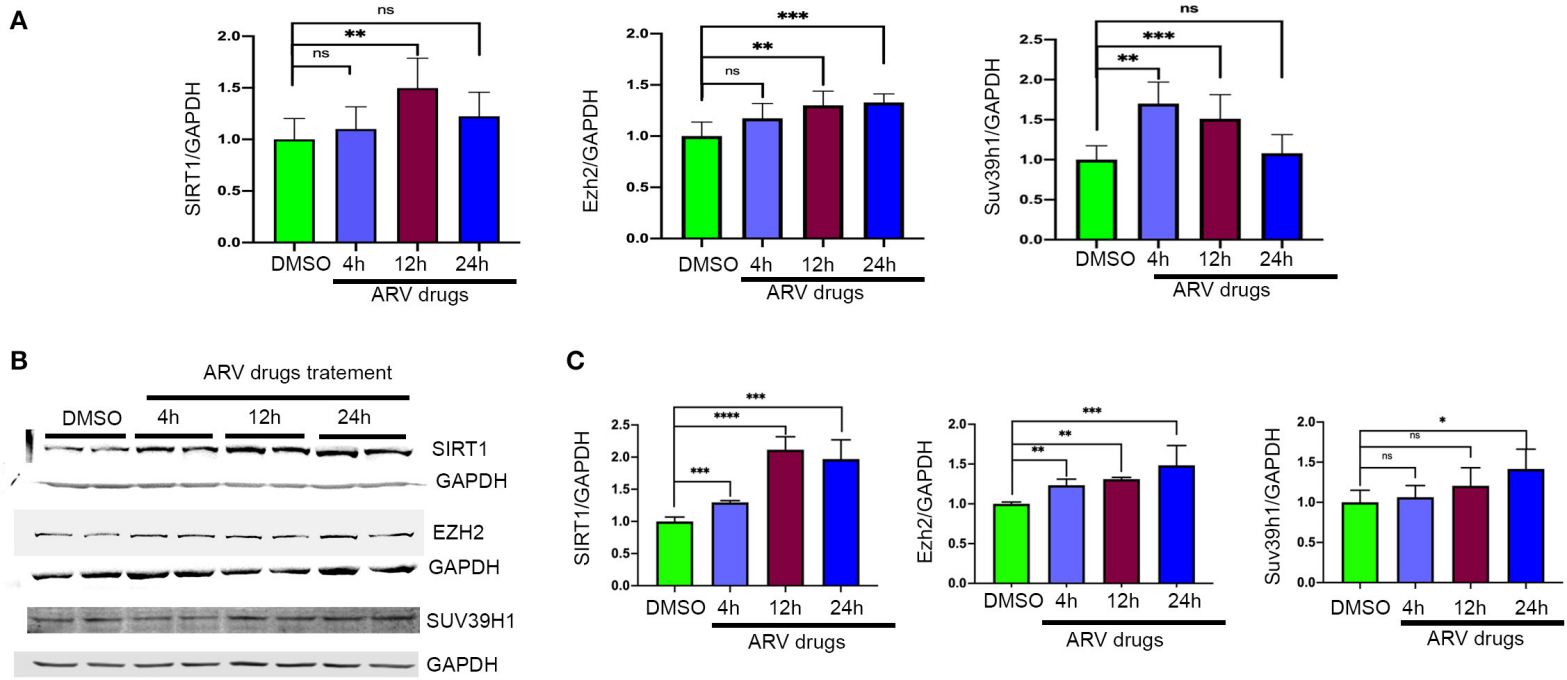
Expression of epigenetic chromatin enzymes affected by ARV drugs treatment. **(A)** Graphs showing mRNA expression of Sirt1, Ezh2, and Suv39h1 in NRVCs treated with drugs. Cells were treated with ARV drugs (5 μM of Ritonavir, Abacavir, Atazanavir and Lamivudine) for different time point (4, 12, and 24 h) and expression was checked by qRT-PCR. **(B,C)** Western blots showing protein expression of SIRT1, SUV39H1, and EZH2 enzymes and graph show quantification. NRVCs were treated with ARV drugs (5 μM of Ritonavir, Abacavir, Atazanavir, and Lamivudine) for 4, 12, and 24 h and expression was checked in total protein lysate by western blot with respective antibody (^*^p < 0.05, ^**^p < 0.01, ^***^p < 0.001, ^****^p < 0.0001, ns, not significant).

**Figure 5:**
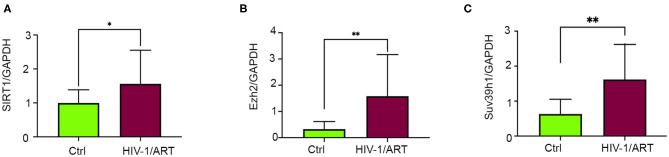
Expression of epigenetic regulating enzymes dysregulated in HIV + patients have history of ART treatment. **(A–C)** Graphs show expression of SIRT1, SUV39H1, and EZH2 enzymes in human cardiac tissue. Total RNA was isolated from the frozen heart tissue and expression was checked by qRT-PCR (^*^p < 0.05, ^**^p < 0.01).

To understand the correlation of ARV drugs and epigenetic changes, we measured the expression of chromatin remodeling enzymes in NRVCs after ARV drugs treatment. NRVCs were treated with ARV drugs for 4, 12, and 24 h, and the expression of chromatin remodeling enzymes was assessed by RT^2^ PCR array profiling. Analysis of the PCR array data showed that out of 84 genes, 50 epigenetic modifying chromatin enzymes were differentially regulated in NRVCs after drug treatment (Table 2). PCR array data analysis represented in clustergrams, scattered plots, and bar graphs (Figures 3A–C) showed that drug treatment differentially regulated the expression of chromatin modifying enzymes in cardiomyocytes. In our western blot data at Figures 1, 2, we found that ARV drugs modify the acetylation as well as methylation of histone 3. Interestingly, in PCR array result we found that the histone deacetylase enzyme SIRT1 (Fold Change (FC) = 1.95; *p* = 0.02) and the methyl transferase enzyme SUV39H1 (FC = 1.36; *p* = 0.01) and Ezh2 (FC = 1.3; *p* = 0.02) were significantly modulated in ARV drug-treated cells compared to control cells (Figures 3A–C). PCR array results for SIRT1, EZH2, and SUV39H1 were validated with qRT-PCR in NRVCs treated with ARV drugs for 4, 12, and 24 h. Expression analysis confirmed that ARV drugs treatment upregulated the expression of SIRT1, SUV39H1, and EZH2 at 12 h post treatment (Figure 4A). We also examined the expression of SIRT1, EZH2, and SUV39H1 at the protein level in NRVCs treated with ARV drugs for 4, 12, and 24 h. Western blot analysis showed that the expression of these epigenetic regulating enzymes was significantly upregulated in drug treated cells (Figures 4B,C). Additionally, we validated our *in vitro* expression data in clinical samples obtained from HIV-1-infected patients treated with ART (Table 3). Total RNA was isolated from heart tissue and the expression of SIRT1, EZH2, and SUV39H1 was measured by qRT-PCR using gene specific primers (Table 1). We used healthy donor human heart tissue for comparison (Table 3). Expression analysis shows that similar to our *in vitro* results, the expression of SIRT1, EZH2, and SUV39H1 was significantly upregulated in HIV+ patients compared to healthy donor samples (Figures 5A–C).

**Table 2:** PCR array analysis of differentially expressed epigenetic chromatin modifying enzymes on ARV drugs treatment.

		**Gene symbol**	**Fold regulation**	***p*-value**
1	ART 4 h	Actb	1.56	1.026
2		Ash2l	1.93	0.013
3		Aurkc	1.75	0.031
4		Crebbp	1.47	0.043
5		Cxxc1	1.33	0.026
6		Dot1l	2.90	0.025
7		Ehmt2	2.60	0.044
8		Hdac10	2.00	0.041
9		Hdac11	2.14	0.020
10		Hdac3	1.43	0.019
11		Hdac4	1.84	0.001
12		Hdac5	2.58	0.047
13		Hdac6	1.66	0.040
14		Hdac7	1.90	0.022
15		Hdac8	1.82	0.013
16		Med24	1.44	0.026
17		Ncor1	1.33	0.036
18		Prmt2	2.25	0.011
19		Prmt6	4.86	0.020
20		Rps6ka5	1.28	0.004
21		Sirt1	1.95	0.026
22		Setd6	1.30	0.046
23		Smyd1	1.50	0.004
24	ART 12 h	Crebbp	1.36	0.026
25		Ep300	1.30	0.019
26		Ezh2	1.28	0.022
27		Mta2	1.31	0.036
28		Ncor1	1.43	0.011
29		Smyd1	1.64	0.027
30		Suv420h1	1.35	0.041
31	ART 24 h	Atf2	1.42	0.032
32		Cdk2	1.34	0.041
33		Edf1	1.52	0.016
34		Ep300	1.70	0.011
35		Ezh2	1.30	0.021
36		Fbxo11	1.44	0.027
37		Ing3	1.29	0.034
38		Med24	1.68	0.030
39		Mta2	1.45	0.027
40		Ncoa6	1.46	0.006
41		Ncor1	1.45	0.048
42		Nek6	1.66	0.019
43		Nsd1	1.68	0.046
44		Prmt1	1.36	0.001
45		Rps6ka5	1.64	0.029
46		Setd6	1.51	0.027
47		Smyd1	1.8	0.005
48		Suv39h1	1.36	0.016
49		Suv420h1	1.87	0.024
50		Ube2b	1.42	0.046

**Table 3:** Patient's information.

**Patients No**	**HIV status**	**Age**	**Race**	**Risk**	**ART**	**Viral load copies/ml**	**CD4**
1	–	50–60	H	na	–	na	na
2	–	50–60	B	na	–	na	na
3	–	50–60	B	na	–	na	na
4	–	50–60	H	na	–	na	na
5	–	40–50	W	na	–	na	na
6	–	60–70	H	na	–	na	na
7	–	60–70	B	na	–	na	na
8	–	30–40	H	na	–	na	na
9	+	60–70	B	ivdu	+	30	137
10	+	60–70	H	sex	+	undetect (<20)	1808
11	+	70–80	W	unknown	+	undetect (<20)	472
12	+	70–80	B	ivdu	+	380	158
13	+	50–60	B	sex	+	undetect (<50)	42
14	+	60–70	B	sex	+	undetect (<20)	55
15	+	NA	NA	na	+	NA	NA
16	+	NA	NA	na	+	NA	NA

### SIRT1 Activity Is Critical for ARV Drugs Mediated Deacetylation of Histone in Cardiomyocytes

**Figure 6:**
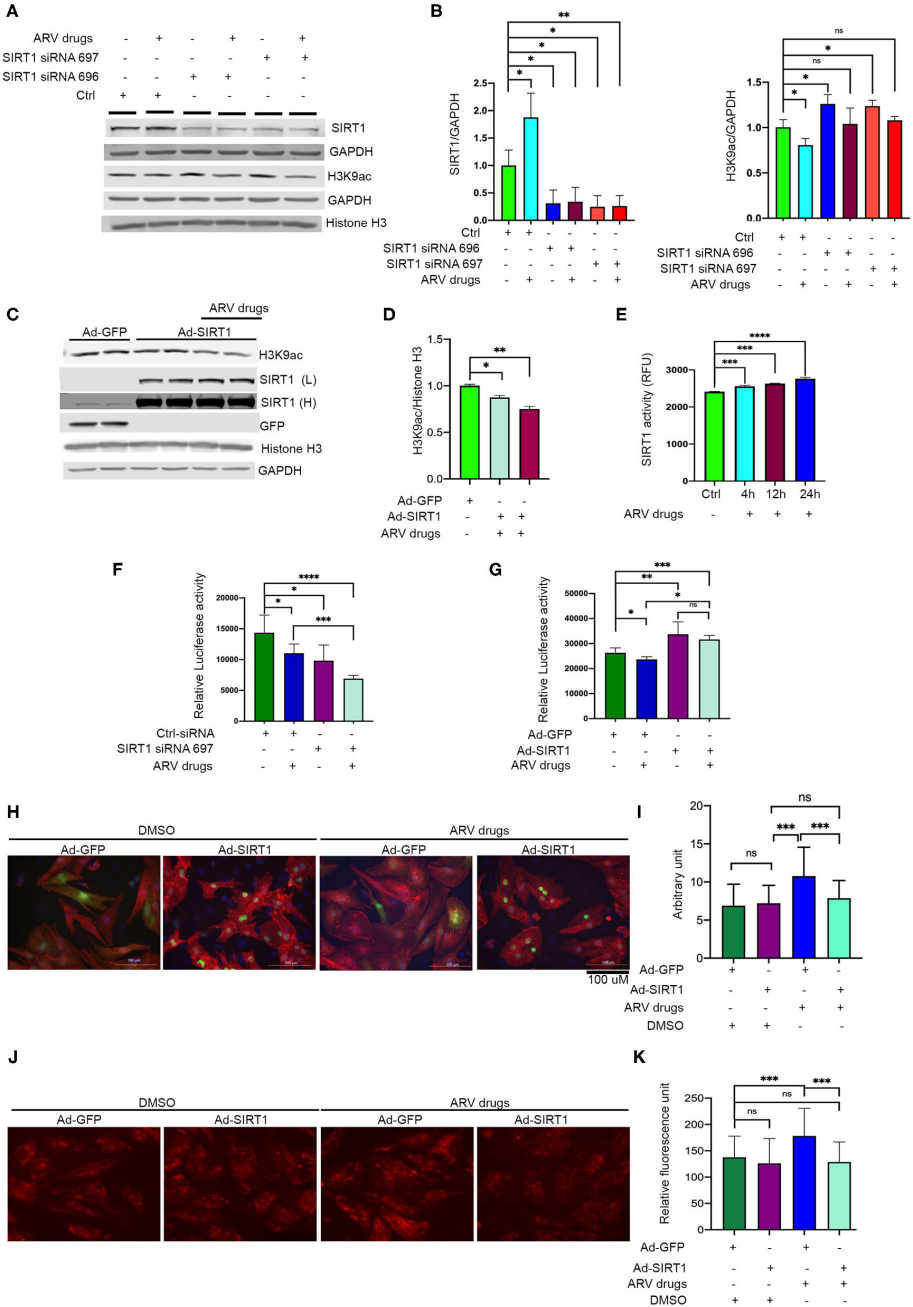
Expression of SIRT1 is critical in ARV drugs mediated modulation of cellular acetylation. **(A,B)** Western blots show that knockdown of SIRT1 upregulate acetylation of histone at H3K9. NRVCs were treated with SIRT1 siRNA for 48 h and followed by ARV drugs treatment (5 μM of Ritonavir, Abacavir, Atazanavir, and Lamivudine) for 12 h. Graph shows quantification of western blot. (^***^p < 0.001; ^**^p < 0.01, ^*^p < 0.05, ns, not significant). **(C,D)** Western blots show that over expression of SIRT1 can significantly decreases the H3K9ac in rat cardiomyocytes. SIRT1 and GFP protein were over expressed in the cardiomyocytes for 48 h by adenoviral transduction and treated with the ARV drugs for another 24 h. Graph shows the quantification of H3K9ac (^*^p < 0.05). **(E)** Drug treatment upregulates the SIRT1 enzyme activity. NRVCs were treated with ARV drugs for 4–24 h and enzyme activity was determined in total protein lysate. (^***^p < 0.001; ^****^p < 0.0001, ns, not significant). **(F)** ARV drugs treatment reduces cellular viability in SIRT1 knockdown cells. NRVCs were treated with SIRT1 siRNA and ARV drugs and viability was measure by CellTiter-Glo (^**^p < 0.01; ^*^p < 0.05, ns, not significant). **(G)** Graph shows that overexpression of SIRT1 improves cellular viability during ARV mediated cellular stress. NRVCs were transfected with adenovirus for 48 h and then treated with ARV drugs for another 24 h. Cellular viability was determined by the CellTiter-Glo (^***^p < 0.001; ^**^p < 0.01, ^*^p < 0.05, ns, not significant). **(H,I)** Representative microscopy images show that ARV drugs treatment induces cellular hypertrophy and SIRT1 over expression significantly reduces the cellular hypertrophy in ARV drugs treated cells (^*^p < 0.05). NRVCs were transfected with adenovirus for 48 h and then treated with ARV drugs for another 24 h. Drug treated cells were fixed with 4% PFA and stained with actinin antibody (red) and DAPI for nucleus. Cell size was determined by Image J software (National Institute of Health, USA). Graph shows the measurement of cell size (^***^p < 0.001, ns, not significant). **(J,K)** Representative images show that ARV drugs treatment induces cellular ROS level and SIRT1 over expression significantly reduces the ROS level of drug treated cells. Graph shows the quantification of ROS. NRVCs were transfected with adenovirus for 48 h and then treated with ARV drugs for another 24 h. ROS level of the cells were determined by DHE staining. Live imaging was done under fluorescence microscope (^***^p < 0.001, ns, not significant).

In our experiments, we found that ARV drugs treatment reduces acetylation of the histone 3 protein at H3K9 and H3K27 (Figures 1A,B). Additionally, our data showed that ARV drugs treatment induces the expression of SIRT1 at the transcriptional as well as translational level (Figures 3C, 4A,B). To explore the role of SIRT1 in ARV drugs mediated changes in histone PTMs, we knocked down SIRT1 expression in NRVCs using siRNA for 48 h and then treated with ARV drugs for 12 h. Expression of H3K9 acetylation was measured by western blotting using total protein lysate. Western blot showed that siRNAs significantly reduced the expression of SIRT1 protein in NRVCs (Figures 6A,B). Interestingly, the acetylation of histone protein at H3K9 was significantly increased in SIRT1 knockdown cells after ART treatment (Figures 6A,B). Concomitantly, we found that overexpression of SIRT1 significantly reduced H3K9 acetylation in both drug treated and untreated cardiomyocytes, which suggests that SIRT1 is a critical regulator of ARV drugs-mediated histone acetylation at H3K9 (Figures 6C,D).

We also assessed the effect of ARV drugs on SIRT1 mediated deacetylase activity in NRVCs. Cells were treated with ARV drugs for 4–24 h, and then deacetylase activity was monitored using whole protein lysate. Deacetylase activity showed that enzyme activity significantly increased after drug treatment (Figure 6E). We also evaluated the effects of drug treatment on cellular viability using NRVCs. Cells were treated with ARV drugs for 24 h and viability was measured using CellTiter-Glo. The viability assay further suggests that drug treatment or knockdown of SIRT1 significantly decreases cellular viability (Figure 6F). Additionally, we found that SIRT1 over expression significantly improves the cellular viability during ARV mediated cellular stress (Figure 6G). Further, we found that SIRT1 expression significantly reduced cellular hypertrophy and ROS production in cardiomyocytes treated with the ARV drugs (Figures 6H–K). Our study suggests that SIRT1 plays a critical role during drug induced cellular toxicity and protects cardiomyocytes from oxidative stress.

### Ezh2 Plays an Important Role in Maintaining the Repressive Histone Marks in Cardiomyocytes

In our study, we found that ARV drugs treatment significantly upregulates the di and trimethylated form of histone (H3K27me2, H3K27me3) along with histone methyl transferase enzyme Ezh2 (Figures 2–4). Further, to elucidate the role of methyl transferase during ARV drugs treatment we knockdown the expression of Ezh2 using siRNA. Western blots show that siRNA treatment significantly reduces the expression of Ezh2 in cardiomyocytes (Supplementary Figures 3A,B). Furthermore, we found that level of H3K27me3 level was significantly reduced in the Ezh2 knockdown cells (Supplementary Figure 3C). Additionally, we checked the cell size of the Ezh2 knockdown cells by microscopy. Microscopy images suggest that Ezh2 knockdown significantly increases the cell size in both DMSO treated as well as in ARV drug treated cells (Supplementary Figures 3D,E) which suggest that Ezh2 plays significant role in regulation of histone methylation and cardiomyocytes function.

## Discussion

Earlier studies suggested that HIV induced cardiomyopathy is one of the leading causes of death in HIV+ patients (51). Based on limited data available worldwide of HIV patients on ART, diastolic dysfunction, and heart failure remain the major causes of comorbidity in the HIV+ population (52). Although application of ART improves the survival of HIV+ patients, the effect of ART on cardiac function is still controversial. In addition, it is not clear how ART and HIV induce cellular and molecular changes in cardiomyocytes, which lead to heart failure (51, 52).

Epigenetic markers control gene-specific expression in cells, which regulates the cellular transcriptome and maintenance of cellular function (32). Distribution patterns of epigenetic markers over the genome helps to determine cell types, and these markers remain mostly stable in mature cells (53). Changes in distribution patterns of epigenetics marks by upstream stress stimuli leads to gene expression modulation and development of disease phenotypes (28, 29, 54). This study explored the molecular mechanism of ARV drugs mediated epigenetic modification of histones along with its epigenetic regulating enzymes. We found that ARV drugs treatment in cardiomyocytes destabilized the active histone marks (H3K9ac and H3K27ac), while promoting the histone repressive marks (H3K9me2, H3K27me2, H3K9me3, and H3K27me3). Similar to our current findings, earlier studies also reported similar epigenetic modifications in mice hearts after pressure overload-induced cardiac hypertrophy, as well as in failing human hearts (31, 32, 55, 56). Those epigenetic modifications of active and repressive histone marks led to changes in pathological gene expression and caused cardiomyocytes remodeling as well as progression of heart failure (57, 58). In our study, we found that cardiomyocytes treated with the ARV drugs leads to production of ROS and cellular hypertrophy which could be one of the mechanisam of ARV drugs medicated induction of cellular toxicity. Studies in clinical samples showed that loss of active gene expression associated with structure and functional changes of heart which may led to progressive heart failure in patients (31, 32). Similarly, we found significant alteration of epigenetic regulatory enzymes in tissue collected from the hearts of HIV patients undergoing ART. This suggests that ART could be one cause of pathological changes in the hearts of PLWH.

During adverse conditions, histone epigenetics dynamically change to regulate DNA template function and fulfill cellular demand. These histone PTMs are regulated by specific epigenetic chromatin enzymes, whose activities are required to make changes at active histone or repressive histone marks. In this study, we found that the expression of the epigenetic enzymes SIRT1, EZH2, and SUV39H1 was upregulated in ARV drugs-treated cardiomyocytes, as well as in heart tissue from HIV+ patients on ART. SIRT1 is an NAD dependent histone deacetylase and promotes transcriptional inactivation by deacetylation of histone marks such as H3K9ac (38, 59). We found that SIRT1 expression and activity increased in cardiomyocytes after ARV drugs treatment. Earlier studies suggested that SIRT1 expression increases during mild oxidative stress as a compensatory mechanisam and gives protection to cells through reduction of ROS production (60). Some studies also suggested that SIRT1 has a cardiac protective role in stress conditions and regulates cellular stress and cell death by allowing deacetylation of H3K9 (37–39, 61). Moreover, SIRT1 protects the heart by regulation of histone acetylation and methylation at H3K9 during ischemic and hypertrophic conditions (39). SIRT1 mediated deacetylation at H3K9 allows the modulation of physiological and pathological gene-specific expression through stress response pathways (62). Similarly, another study showed that SIRT1 agonism can protect cells by inhibition of inflammatory cytokine production, and reduces cellular oxidative stress by modulation of forkhead box O1 (Foxo1) (38). Additionally, SIRT1 can attenuate apoptosis in cardiomyocytes by preventing induction of caspase-3, as well as inhibition of autophagy by deacetylating major autophagy regulatory factors such as Atg5, Atg7, and Atg8 (63, 64). In our study we found that increased expression of SIRT1 can reduces ROS level of ARV drugs treated cells and improve cellular viability as well. Since, SIRT1 plays a cardioprotective role in response to oxidative stress in the cardiomyocytes, this may explain why upregulation of SIRT1 provides protection to cells during ART induced oxidative stress.

Moreover, ARV drugs treated cardiomyocytes or heart tissue from ART treated patients showed upregulation of the H3K9 and H3K27 methyl-transferase enzymes, EZH2 and SUV39H1, respectively. EZH2 is an enzymatic subunit of polycomb repressive complex 2, and is the only known H3K27 methyltransferase (65). A previous study suggested that it is essential to maintain EZH2 expression levels in cardiomyocytes to regulate cellular homeostasis and physiological gene expression. It is also reported that a hypertrophic response can promote EZH2 expression levels in the heart (66). In our current study, we found that Ezh2 protein required to maintain the cellular repressive histone marks (H3K27me3) and knockdown of Ezh2 causes cellular hypertrophy. The molecular mechanism driving EZH2 interaction in cardiomyocytes is not clear, however, TAC induced cardiac hypertrophic responses in mice showed that EZH2 interacts with the primary microRNA-208b to regulate the expression of antisense β-MHC and α-MHC (67, 68). Ezh1 and Ezh2 expression changes during heart development and regulates cellular gene expression by turn on and turn off mechanisam on responsive gene through switching from mono to di and trimethylation of histone (67, 69, 70). In this study, we found that another epigenetic chromatin enzyme, SUV39H1, was upregulated due to drug treatment. SUV39H1 is known to differentially regulated, and its increased expression is reported to be associated with the development of cardiac hypertrophy (71). The molecular mechanism of SUV39h1-mediated regulation of cardiomyocyte pathology in hypertrophic conditions is not clear; however, a previous study showed that kindlin-2 can interact with SUV39H1 and recruit the complex to the GATA4 gene promoter to suppress GATA4 transcription by bi- and trimethylation at H3K9 (72). Interestingly, some studies suggest that SIRT1 and SUV39H1 regulates each other expression and function through deacetylation and methylation, respectively, depending on the cellular stress condition (38, 40). These data support that ARV drugs treatment of cardiomyocytes dysregulates the expression of epigenetic chromatin modifying enzymes and their downstream targets in histones, which triggers repression histone marks, as reported in cardiac tissue of heart failure patients.

## Conclusion

Taken together, our data shows that combined ARV drugs treatment induces cardiotoxicity, which may lead to the development of cardiac dysfunction in HIV patients. ARV drugs-mediated cellular stress causes aberration of PTM at histones, which suppresses active histone marks and promotes inactivation of histone marks through the modulation of the expression and activity of chromatin modifying enzymes. Additionally, we found that SIRT1 plays a critical role in the regulation of acetylation levels at histone proteins, and may give protection to cardiomyocytes and the heart during ARV drugs-mediated stress.

## Data Availability Statement

The original contributions presented in the study are included in the article/Supplementary Material, further inquiries can be directed to the corresponding author/s.

## Ethics Statement

All experiments were performed in accordance with relevant guidelines and regulations, and this study was reviewed and approved by the University of Central Florida Institutional review board (IRB). All patient samples were obtained under informed consent and approved by local IRB. Written informed consent for cardiac tissues was obtained and is maintained by the members of the National NeuroAIDS Tissue Consortium (NNTC) under local IRB approved protocol. De-identified samples were provided by the NNTC for this study. The patients/participants provided their written informed consent to participate in this study. The animal study was reviewed and approved by IACUC.

## Author Contributions

SK, MG, PD, JR, and JJ design the experiments. SK, AM, DG, and MG perform the experiments. SK and MG analyze the data. PD, JR, JJ, SE, and MG participate analysis of data and critical evaluation of manuscript. All authors contributed to the article and approved the submitted version.

## Conflict of Interest

The authors declare that the research was conducted in the absence of any commercial or financial relationships that could be construed as a potential conflict of interest.

## Supplementary Material

Supplementary Figure 1 ARV drug treatment causes upregulation of dimethylated histone in cardiomyocytes. **(A)** Western blot data show that ARV drugs treatment significantly upregulates dimethylated histone (H2K9me2 and H3K27 me2), but monomethylated form of histone did not change (H2K9me1 and H3K27 me1). NRVCs were treated with ARV drugs (5 μM of Ritonavir, Abacavir, Atazanavir, and Lamivudine) for 4–24 h and western blot was performed with total protein lysate using antibodies specific to mono and dimethylation of histone. **(B)** Graph shows quantification of western blots (^**^*p* < 0.01; ^*^*p* < 0.05).

Supplementary Figure 2 ARV drugs treatment induces cellular hypertrophy and ROS production in cardiomyocytes. **(A–C)** Representative images show that ARV drug treatment increases the cell size in cardiomyocytes. Graph shows the quantification of cell size. NRVCs were treated with ARV drugs (5 μM of Ritonavir, Abacavir, Atazanavir, and Lamivudine) for 24 h and cells were fixed with 4% PFA. Fixed cells were stained with actinin antibody (red) and DAPI for the nucleus (blue). **(D–F)** Representative images and graph show that ARV drug treatment induces ROS production in cardiomyocytes. Cells were treated with ARV drugs (5 μM of Ritonavir, Abacavir, Atazanavir, and Lamivudine) for 24 h and stained with DHE. Live cell imaging was done with the help of fluorescence microscopy. Graph shows the quantification of DHE staining.

Supplementary Figure 3 Ezh2 is critical to maintain the histone repressive marks of histone. **(A–C)** western blots show the expression of Ezh2 and H3K27m3 in cardiomyocytes treated with Ezh2 siRNA. NRVCs were treated with Ezh2 siRNA for 48 h and then treated with ARV drugs for another 12 h (5 μM of Ritonavir, Abacavir, Atazanavir, and Lamivudine). Western blot was performed with total protein lysate. Graph shows the quantification of western blots. **(D,E)** Representative microscopy images show that knockdown of Ezh2 induces cellular hypertrophy. NRVCs were transfected with siRNAs for 48 h and then treated with ARV drugs for another 24 h. Drug treated cells were fixed with 4% PFA and stained with actinin antibody (red), Ezh2 antibody (green) and DAPI for nucleus. Cell size was determined by Image J software. Graph shows the measurement of cell size (^***^*p* < 0.001, ^**^*p* < 0.01, ^*^*p* < 0.05).
