# Supplementary figures and images for: Antiretroviral Drugs Regulate Epigenetic Modification of Cardiac Cells Through Modulation of H3K9 and H3K27 Acetylation
Source: Front Cardiovasc Med. 2021 Apr 9;8:634774. doi: 10.3389/fcvm.2021.634774 (PMC8062764; doi:10.3389/fcvm.2021.634774)

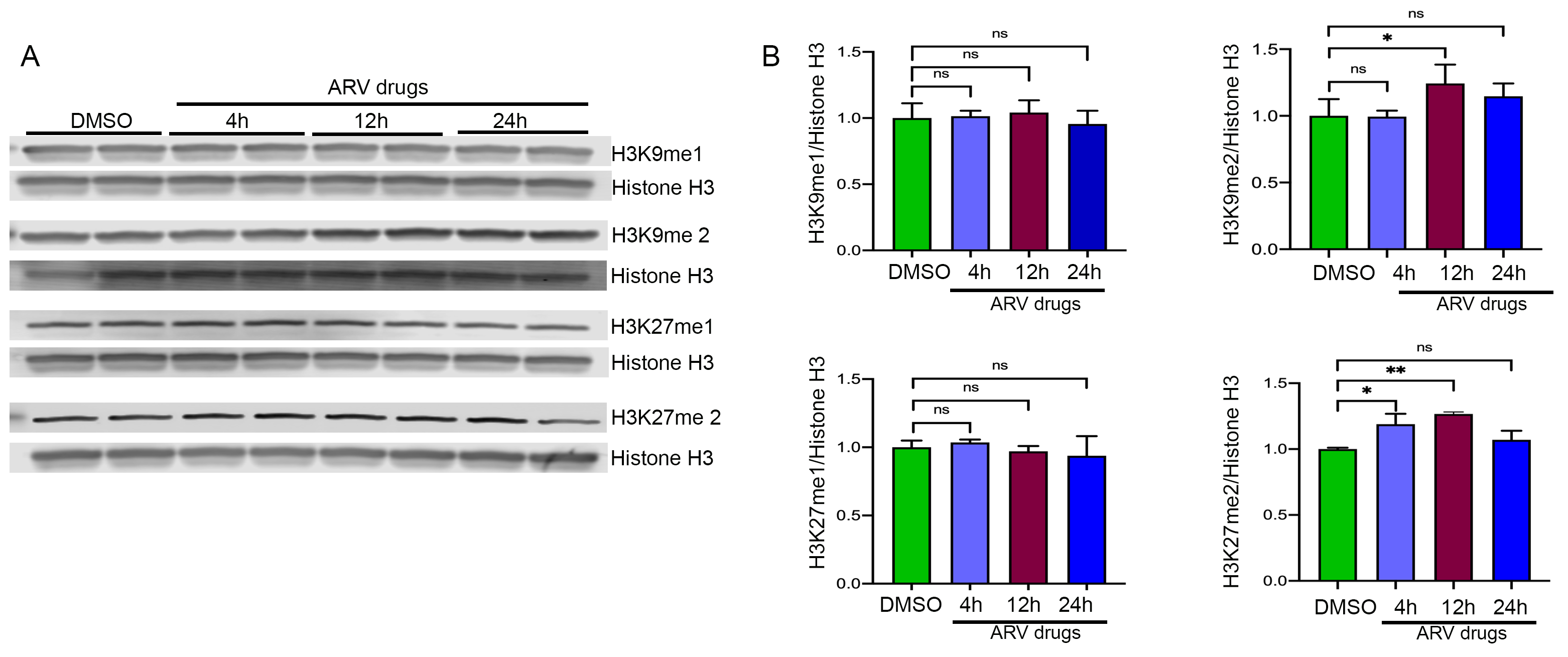

Supplement: Supplementary Figure 1 — ARV drug treatment causes upregulation of dimethylated histone in cardiomyocytes. (A) Western blot data show that ARV drugs treatment significantly upregulates dimethylated histone (H2K9me2 and H3K27 me2), but monomethylated form of histone did not change (H2K9me1 and H3K27 me1). NRVCs were treated with ARV drugs (5 μM of Ritonavir, Abacavir, Atazanavir, and Lamivudine) for 4–24 h and western blot was performed with total protein lysate using antibodies specific to mono and dimethylation of histone. (B) Graph shows quantification of western blots (**p < 0.01; *p < 0.05). [file Image_1.TIF]

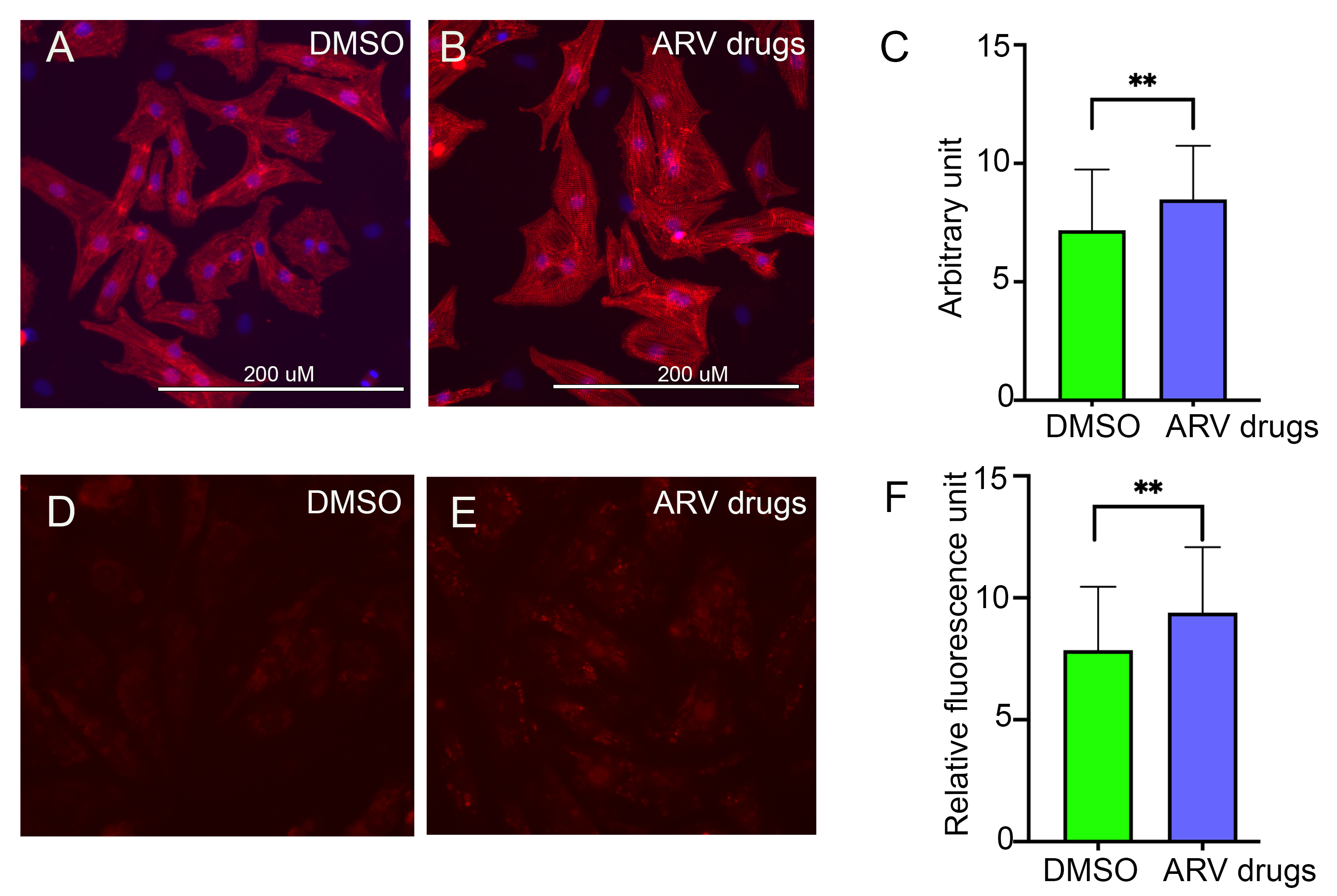

Supplement: Supplementary Figure 2 — ARV drugs treatment induces cellular hypertrophy and ROS production in cardiomyocytes. (A–C) Representative images show that ARV drug treatment increases the cell size in cardiomyocytes. Graph shows the quantification of cell size. NRVCs were treated with ARV drugs (5 μM of Ritonavir, Abacavir, Atazanavir, and Lamivudine) for 24 h and cells were fixed with 4% PFA. Fixed cells were stained with actinin antibody (red) and DAPI for the nucleus (blue). (D–F) Representative images and graph show that ARV drug treatment induces ROS production in cardiomyocytes. Cells were treated with ARV drugs (5 μM of Ritonavir, Abacavir, Atazanavir, and Lamivudine) for 24 h and stained with DHE. Live cell imaging was done with the help of fluorescence microscopy. Graph shows the quantification of DHE staining. [file Image_2.TIF]

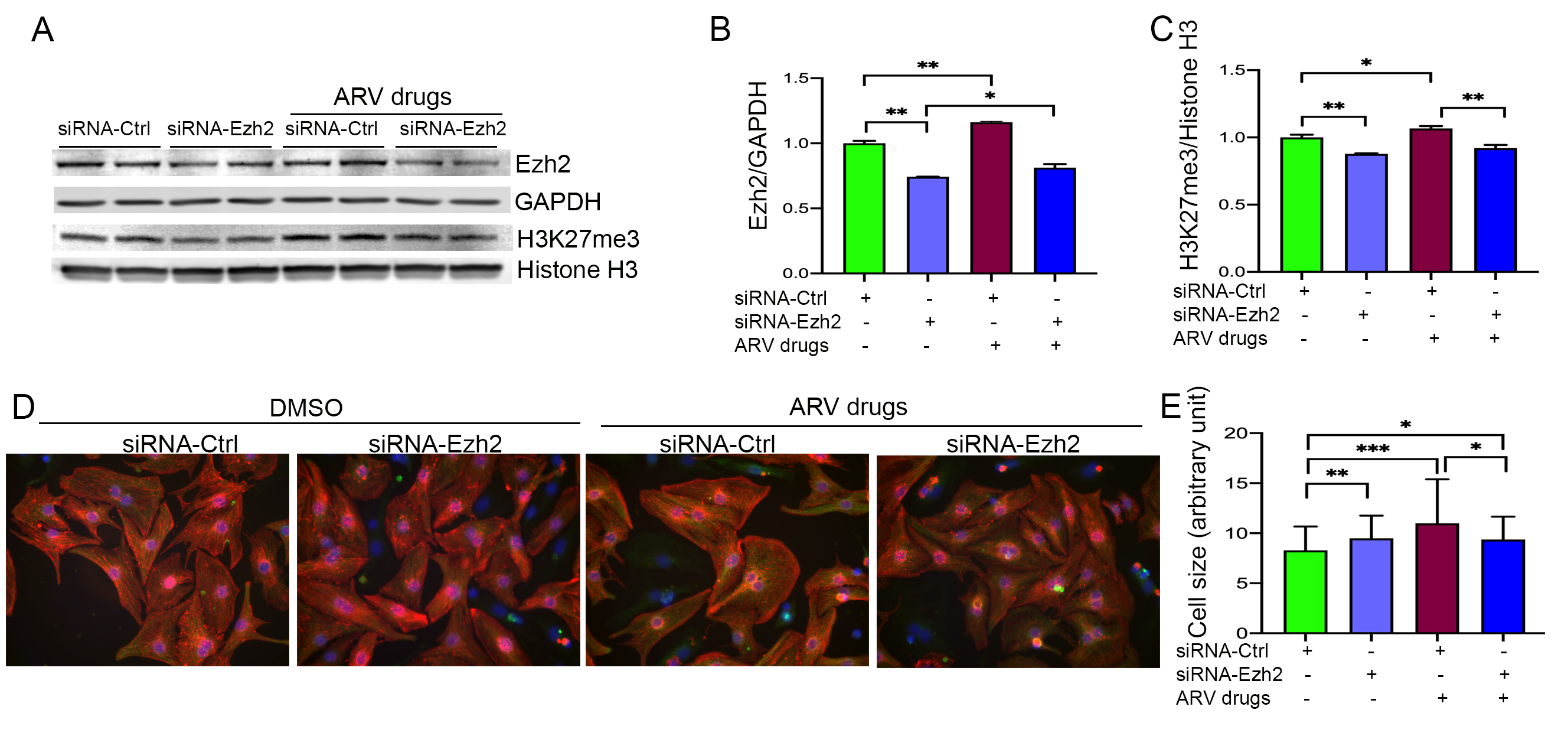

Supplement: Supplementary Figure 3 — Ezh2 is critical to maintain the histone repressive marks of histone. (A–C) western blots show the expression of Ezh2 and H3K27m3 in cardiomyocytes treated with Ezh2 siRNA. NRVCs were treated with Ezh2 siRNA for 48 h and then treated with ARV drugs for another 12 h (5 μM of Ritonavir, Abacavir, Atazanavir, and Lamivudine). Western blot was performed with total protein lysate. Graph shows the quantification of western blots. (D,E) Representative microscopy images show that knockdown of Ezh2 induces cellular hypertrophy. NRVCs were transfected with siRNAs for 48 h and then treated with ARV drugs for another 24 h. Drug treated cells were fixed with 4% PFA and stained with actinin antibody (red), Ezh2 antibody (green) and DAPI for nucleus. Cell size was determined by Image J software. Graph shows the measurement of cell size (***p < 0.001, **p < 0.01, *p < 0.05). [file Image_3.TIF]
